# Quality parameters of chicken breast meat affected by carcass scalding conditions

**DOI:** 10.5713/ajas.18.0692

**Published:** 2019-02-07

**Authors:** Rosana Aparecida da Silva-Buzanello, Alexia Francielli Schuch, André Wilhan Gasparin, Alex Sanches Torquato, Fernando Reinoldo Scremin, Cristiane Canan, Adriana Lourenço Soares

**Affiliations:** 1 Department of Food Science and Technology, Universidade Estadual de Londrina, Celso Garcia Cid, PR 445 Km 380, Londrina, PR, ZIP 86057-970, Brazil; 2 Post-Graduation Program of Food Technology (PPGTA), Universidade Tecnológica Federal do Paraná (UTFPR), Av. Brazil 4232, Medianeira, PR, ZIP 85884-000, Brazil; 3 Cooperativa Agroindustrial Lar, Slaughter Supervision, Matelândia, PR, ZIP 85887-000, Brazil; 4 Chemistry Department, Universidade Tecnológica Federal do Paraná (UTFPR), Av. Brazil 4232, Medianeira, PR, ZIP 85884-000, Brazil; 5 Chemistry Department, Universidade Federal da Integração Latino-Americana (UNILA), Av. Tancredo Neves 6731, Foz do Iguaçu, PR, ZIP 85867-900, Brazil

**Keywords:** Emulsification Capacity, Fatty Acid Profile, Protein Denaturation, Protein Secondary Structure

## Abstract

**Objective:**

The influence of broiler carcass scalding conditions on chicken breast meat quality parameters was investigated.

**Methods:**

Two hundred and seventy Cobb broiler chickens from 42 to 48 days old were slaughtered according to the standard industry practice and scalded in five temperature/time combinations—T_1_, 54°C/210 s; T_2_, 55°C/180 s; T_3_, 56°C/150 s; T_4_, 57°C/120 s; T_5_, 58°C/90 s.

**Results:**

Scalding temperature increase resulted in higher values of external and ventral lightness and in protein functionality reduction—determined by emulsification capacity and protein denaturation—in chicken breast fillets 24 h *post-mortem*. Protein secondary structures had conformational changes, with a decrease of the α-helix and an increase of the β-sheet and β-turn proportions, mainly in T_1_ and T_5_ samples, determined by Fourier-transform infrared spectroscopy in an attenuated reflectance mode analysis. The chemical composition, pH, water holding capacity and Warner-Bratzler shear force did not differ among the treatments. In the fatty acid profile, the 18:1n-9 was lower in T_5_, which suggested that the high scalding-temperature could have caused the lipid oxidation. The values of the polyunsaturated fatty acids (PUFA), such as 22:2, 20:4n-6, and 22:6n-3, were highest in the T_5_, thus being related to the phospholipid cellular membrane collapse in this experimental condition and subsequent release of these PUFA.

**Conclusion:**

Intermediate scalding-parameters avoided the negative changes in the chicken meat quality.

## INTRODUCTION

In a commercial poultry slaughterhouse, the broiler carcasses are immediately submitted to scalding after stunning and exsanguination. In this step, the carcasses are typically immersed in a hot water tank with both temperature and time controlled, to facilitate the defeathering as much as 80% less force [1,2].

This process can be classified as hard or soft in relation to the time and temperature combination of scalding. In hard-scald systems, the water temperature varies from 60°C to 66°C, and the time varies from 45 to 90 s. Less common systems can use water temperatures from 54°C to 58°C and time from 60 to 120 s or a slightly higher temperature from 60°C to 63°C for 15 to 30 s [3]. Temperatures varying from 51°C to 54°C with higher immersion times (from 120 to 210 s) are the conditions applied in the soft-scald method [4,5]. When hard scalding is applied—warmer temperature and short time—it also removes the outermost epidermal or cuticle layer, resulting in white skin on the carcasses [6,7].

Although the scalding procedure must be selected to provide effective defeathering, this operational step should not affect the carcass quality [1]. Brazil’s broiler slaughterhouses have reported problems with the use of hard scalding, resulting in carcasses with superficial burns. This alteration is named overscalding and results in a banding appearance on the surface of breast meat [7]. Some studies have demonstrated that the scalding procedure is responsible for changes in the broiler muscles, affecting the meat quality. Bowker et al [6] studied the effect of scalding and chilling methods in chicken muscles quality, and they observed that hard-scalding resulted in lower myofibril protein solubility and higher degradation in breast fillets deboned 4 h *post-mortem*. When the protein is submitted to heating, its conformational structure can be modified [8], and the alteration of the protein native structure can affect their functional properties. Changes in the chicken meat properties, mainly in the protein stability, can harm its application in industrialized products and, consequently, result in higher cooking losses and lower yields. Additionally, the use of hard scalding has been related to the decrease of the carcass yield due, mainly, to lipid losses by liquefaction [2,3]. However, the evaluation of the fatty acid profile of chicken meat from broilers scalded at different conditions has not yet been reported.

Current studies that have evaluated the influence of the scalding procedure on the chicken meat quality compared just two scalding conditions—hard and soft scalding—and other combinations of time vs temperature were not reported. The evaluation of different time and temperature combinations in the scalding procedure would allow identification its effect in the broiler muscles and determination of the ideal conditions. Thus, the objective of this study was to evaluate the influence of broiler carcass scalding conditions (temperature vs time) on chicken breast meat quality parameters.

## MATERIALS AND METHODS

### Sample preparation

The experiments were performed in the summer of 2017 at a commercial processing plant located in the state of Paraná, Brazil. The commercial plant slaughters 340,000 broilers/d in two lines at a rate of 12,000 broilers/h, operating in three shifts. Two hundred and seventy Cobb broiler chickens of both genders from 42 to 48 days old and with weights of 2.64±0.32 kg were slaughtered according to the standard industry practice: electrical stunning, bleeding, scalding, defeathering, evisceration and carcass water-cooling. The Ethical Committee of Animal Research of the State University of Londrina, Brazil approved this research (protocol number: 3158.2016.57).

### Experimental design

A randomized block design was applied in this study using three different lots of birds and each lot came from the same farm and cargo truck. In all lots, a hundred and fifty birds were unloaded from the cargo truck, identified and weighted to posteriorly determine the carcass and breast yields. The birds were randomly divided into groups for five different scalding treatments, which varied the temperature vs time of the procedure—T_1_: 54°C/210 s; T_2_: 55°C/180 s; T_3_: 56°C/150 s; T_4_: 57°C/120 s; T_5_: 58°C/90 s. The scalding parameters of the treatments were defined in preliminary studies, starting from the usual condition used by commercial slaughterhouse (58°C/90 s) reducing the temperature and increasing the time proportionately, and observing the defeatering efficiency. All treatments used promoted efficient feathers and epidermal layer removal by carcasses visual observation in the production line.

The scalding was applied after exsanguination by immersion using a tank with dimensions of 12,000×1,200 mm (length× width) and 12 m^3^ of water capacity, scalding three hundred birds at a time.

After scalding, 18 carcasses/treatment/block (n = 270) were collected and the carcasses were reweighted after evisceration to determine the eviscerated carcass yield.

After water-cooling, approximately 1.5 h *post-mortem*, the carcasses were dripped and reweighed to determine the chilled carcasses yield. The breast meat (deboned skinless, combined left, and right fillets) was removed from the carcass and weighed to determine the breast yields. The chicken breast fillets (*Pectoralis major* m.) from left sides were collected and stored in a cold room at 5°C and then submitted to analysis of color (L*, a*, and b*), pH, Warner-Bratzler (WB) shear force, water holding capacity (WHC), emulsification capacity (EC), protein denaturation (PD), fatty acid profile and Fourier-transform infrared spectroscopy in an attenuated reflectance mode (FTIR-ATR) at 24 h *post-mortem*. The chicken breasts from the right sides were frozen using tunnel freezing (−35°C) and maintained at −18°C until determination of moisture, protein, lipid and moisture/protein ratio.

### Analytical determinations

#### Color measurements

The color measurements were performed on three different sites—the proximal and distal extremity of the muscle and the medial side at the halfway point between the extremities [9]. The color parameters L* (lightness), a* (redness), and b* (yellowness) (CIELAB, Commission Internationale de L’éclairage color system) were evaluated using a Minolta CR400 colorimeter (Osaka, Japan) with a D65 illuminant and a 10° standard observer on the external and underside surface (ventral) of the intact skinless breast muscles 24 h *post-mortem*.

#### pH measurements

The pH was measured in triplicate by inserting electrodes into the breast muscle using a pH meter (HI 99163, Hanna Instruments Brasil, Barueri, SP, Brazil), reported by Olivo et al [9].

#### Warner-Bratzler shear force

Raw samples of chicken breast fillets were cut into pieces of 1 cm×1 cm×2 cm (height×width ×length), with length following the fiber direction. The samples were sheared in triplicate using a WB shear-type blade with a 4 mm/s test speed coupled with a TA-HD plus texture analyzer (Stable Micro Systems, Surrey, UK) fitted with a 5-kg load cell. The blade cut the across fibers, and the maximum force required to cut was measured and expressed in Newton (N).

#### Water holding capacity

This parameter was determined in triplicate according to Hammn [10] with modifications. The samples were weighed (2.0±0.10 g), placed between two filter papers and left under a 10.0 kg weight for 5 min. The samples were reweighed, and the WHC was determined via the exuded water weight using Equation 1:

(1) $$WHC=100-\left(\frac{i-f}{i}\times 100\right)$$

where *i* is the initial weight and *f* is the final weight.

#### Emulsification capacity

To determine the EC adaptations the methodologies proposed by Olivo and Shimokomaki [11] and Qiao et al [12] were employed. A 1 g sample of ground chicken breast meat was blended with 3 mL of cold 2.5% NaCl solution at high speed for 2 min. The homogenate was maintained under high speed blending in an ice bath, and soybean oil mixed with 0.3 g/L of a red colorant (Sudan III, S4131, Sigma Aldrich, Milwaukee, WI, USA) was dripped using a burette at a constant rate of 0.5 mL/min. The oil was added until the solution was observed to change phases, evidenced by a viscosity change, a darkening color, and an audible change in motor speed. The total amount of oil used was recorded and used to express the EC as the amount of oil (mL) needed to affect the phase change.

#### Protein denaturation

The measurement of soluble PD in the chicken breast meat was carried out according to the methodology described by Swatland [13] with modifications. A 5 g of ground chicken breast meat was added to 15 mL of distilled water and homogenized. The mixture was centrifuged at 4,000×g for 20 min at 25°C. The supernatant was filtered, and an aliquot of 1 mL was transferred to a tube containing 5 mL of 0.2 mol/L citrate-phosphate buffer at pH 4.6. A blank was prepared with 1 mL of filtrate and 5.0 mL of distilled water. The sample transmittance at 600 nm was recorded against white in a UV-Vis spectrophotometer (Perkin Elmer, Lambda XLS- Beaconsfield, UK). The PD was expressed in transmittance %.

#### Fatty acid profile

Lipid extraction was performed following the methodology of Bligh and Dyer [14] with modifications. A 15 g of crushed chicken breast meat samples with the moisture corrected to 80% were homogenized in methanol (30 mL) and chloroform (15 mL) for 5 min. Chloroform (15 mL) was added to the mixture, and the homogenization continued for 2 min. Distilled water (15 mL) was then added to the mixture, and the homogenization continued for 5 min. The homogenate obtained was filtered and transferred to a separation funnel. A saturated solution of NaCl equivalent to 1/5 of the volume of the filtrate was added to the separation funnel. After phase separation, the lower phase containing chloroform and fatty matter was collected, and the solvent was evaporated in a rotatory evaporator (801, Fisatom, São Paulo, SP, Brazil) with the bath at 33°C±2°C.

Method 5509 of the International Organization for Standardization [15] was used for fatty acid transesterification. An aliquot of 200 mg of extracted fatty acid matter was transferred to a 10 mL tube with a screw cap; then, 2 mL of n-heptane was added; and the mixture was stirred until complete dissolution of the fatty matter. A 2 mL aliquot of 2 mol/L KOH/methanol was added to the mixture, and it was submitted to vigorous stirring to obtain a slightly turbid solution. After phase separation, the superior phase containing n-heptane and fatty acid methyl esters (FAME) was collected, transferred into 2-mL vials, and stored in a freezer (−18°C) for later chromatographic analyses.

FAMEs were analyzed by gas chromatography (Perkin Elmer, Clarus 680 GC, Waltham, MA, USA) with flame ionization detection and a fused silica capillary column (100 m× 0.25 mm) with 0.25 μm of a cyanopropyl polysiloxane CP 7420 stationary phase. The carrier gas was helium (1.1 mL/min), and the flame gases were hydrogen and synthetic air (40 and 400 mL/min, respectively). The split was 1:100, and the column temperature was set to 80°C for 1 min, ramped at 20°C/min to 160°C, then ramped at 1 °C/min to 198°C, and finally ramped at 5°C/min to 250°C, where it was held for 1.6 min. The injector and detector temperatures were set at 240°C and 250°C, respectively. For peak area determination, an Integrator-Processor CG-300 (Scientific Instruments CG, São Paulo, SP, Brazil) was used, and peaks were identified by comparison of the retention times with FAME standards (Sigma, USA).

#### Fourier transform infrared spectroscopy in an attenuated reflectance mode

Possible additional information about protein structural changes influenced by scalding treatments was obtained by FTIR spectroscopy (Frontier PerkinElmer, Beaconsfield, UK) in attenuated reflectance mode (ATR). Cranial portions of chicken breast meat samples were freeze dried (Labconco, FreeZone 6L, Kansas, MO, USA) at 40°C and 0.4 mBar for 48 hours, and the FTIR-ATR spectra were obtained in the wavenumber range from 600 to 4,000 cm^−1^ during 10 scans with 4 cm^−1^ resolution. A straight baseline passing through the ordinates at 1,720 and 1,570 cm^−1^ (amid I band) was adjusted as an additional parameter to obtain the best fit. The deconvolution of this wavenumber range was determined using a Gaussian curve fit. Considering that all of the main secondary structural elements constituted a linear sum in the proteins and the percentage of each one was related to the spectral intensity, the β-sheet, α-helix, and β-turn portions were determined [16].

### Chemical composition

A FOSS FoodScan near-infrared spectrophotometer was used to determine the moisture, protein, lipid content, and the moisture/protein ratio of the chicken breast meat from the right side, according to the AOAC procedure [17]. The samples were previously unfrozen under refrigeration (5°C) and crushed in a cutter (K55, FOSS, Hillerød, Denmark) before analysis. The equipment was previously calibrated according to fabricant recommendations using the methodologies of the AOAC [18].

### Statistical analysis

Analysis of variance and the Tukey test at a significance level of 5% (p≤0.05) were used to evaluate the data obtained. In these analyses, the treatments and blocks were considered as causes of variation. Statistics analyses were done using the Statistica 7.0 software (Statsoft Inc., Tulsa, OK, USA, 2004).

## RESULTS AND DISCUSSION

### Physicochemical properties

Color is one of the most important quality attributes, and is related to the functional properties of the meat, directly impacting consumer product selection and cooked product appearance [19].

Scald-burn has already shown influence on the external luminosity of chicken breast fillets. Therefore, it has been recommended that instrumental color measure should be conducted in the ventral portions of the fillets [11]. In the present study, the color measure was recorded on both fillets portions (external and ventral). This methodology was employed to verify whether the scalding could also affect the ventral portion of the fillets.

The values of L*, a*, and b* of the ventral portion and L* and a* of the external portion were different among the treatments (p≤0.05) (Table 1). For T_1_ (54°C/210 s) and T_5_ (58°C/90 s), the L* values varied from 53.72 to 57.55 for the ventral measurement, and from 60.11 and 62.69 for the external measurement, respectively. These results confirmed that the use of higher scalding temperatures can also exert influence on the ventral portion of chicken breast fillets. In general, when the scalding temperature was increased the samples exhibited a paler appearance that could have resulted from PD caused by heating.

The L* value was used to classify the chicken breast meat color as pale (L*>53), dark (L*<46), and normal (46<L*<53) [12]. Although the L* values increased with the higher scalding temperatures, all samples in the present study could be classified as pale, independently of the scalding treatment. The pH values varied from 5.84 to 5.89 and they did not differ among the treatments (p>0.05). These pH values were considered normal in all assays (pH>5.80) [20]. A higher incidence of pale chicken breast meat with normal pH has been reported by Brazilian slaughterhouses, and the cause has not been fully elucidated [21].

Although the scalding treatments affected the meat color (external and ventral), this modification did not influence the parameters of WHC and WB shear force (p>0.05) (Table 1). The obtained values varied from 68.30% to 68.91% and from 9.56 to 10.16 N, respectively. WHC values were similar to reported in literature for chicken breast meat [20–22]. Bowker et al [6] also observed no difference in the WB shear force values in chicken breast meat samples from broilers submitted to hard or soft-scalding methods.

The chemical composition of chicken breast meat did not differ (p>0.05) among the applied scalding treatments. Past literature that studied the effect of hard and soft scald-method has reported upon the influence of the scalding parameters on chicken skin composition, but no differing values were found for the chicken breast meat [3].

The lipid content of chicken breast meat varied from 1.91 to 2.11 g/100 g (Table 1). The *pectoralis major* muscle is one of the chicken meat cuts that exhibit the lowest lipid content, varying from 1% to 3% for skinless cuts [23], corroborating the results obtained in the present study. The values of the protein and moisture content and moisture/protein ratios were in agreement with the recommendation by the Brazilian legislation that presents the parameters for evaluation of the total water content in cooled and frozen chicken meat cuts [24].

In contrast to results reported by Buhr et al [3], the yield values of the eviscerated carcass, chilled carcass and deboned chicken breast (Table 1) were not different among the scalding parameters applied (p>0.05), varying from 74.17% to 74.84%, from 79.14% to 80.70%, and from 24.03% to 24.34%, respectively.

### Functionality and secondary structure of the protein

Scalding effects on meat quality would be mediated by myofibril and sarcoplasmic proteins changes [6] that would affect the protein functional properties. In the present study, the protein functionality of the chicken breast meat was evaluated by PD (%) and EC (expressed in x mL of oil for 1 g of sample) measurements (Table 1). The EC values were higher (p≤0.05) in T_1_ (15.44), T_2_ (15.72), and T_3_ (15.59) than in T_4_ (14.42) and T_5_ (14.46). The inverse was observed for PD—T_1_ (26.02%), T_2_ (25.00%), T_3_ (26.21%), T_4_ (31.80%) and T_5_ (32.59%). The results confirmed that increasing of the scalding temperature from 54°C to 58°C, applied for their corresponding times, demonstrated a negative impact on protein functionality. Heat is able to cause PD, resulting in conformational changes. A decrease of the α-helix and an increase of the surface hydrophobicity of the miofibrilar proteins were related to the temperature increasing by approximately 30°C [8]. This kind of alteration could be sufficient to modify the protein functional properties and can justify the effects observed in the present study.

**Figure 1:**
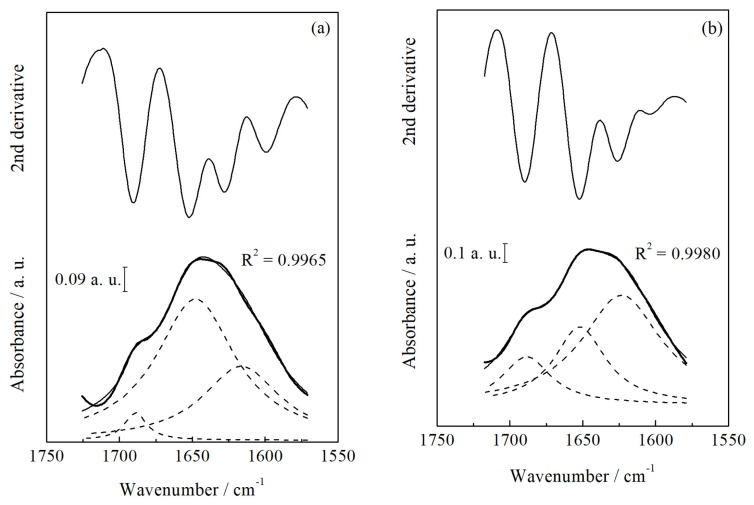
Fourier transform infrared spectroscopy in an attenuated reflectance mode (FTIR-ATR) deconvoluted spectra of chicken breast meat samples from broiler carcass scalding at 56°C/150 s (T3) (a) and at 58°C/90 s (T5) (b) in a wavenumber range from 1,720 to 1,570 cm^−1^ corresponding the amide I band. Original FTIR-ATR spectrum (thick solid line); Gaussian curve-fit (solid line); peaks from deconvolution analysis (dashed lines).

Fourier-transform infrared spectroscopy in an attenuated reflectance mode (FTIR-ATR) was carried out to relate the effects of scalding parameters to changes in the protein secondary structure conformation. Figure 1 shows the amide I band—corresponding to wavenumbers from 1,720 to 1,570 cm^−1^, which represents the C=O and a small amount of the C–N stretching vibration—from the T_3_ and T_5_ samples and the deconvolution of the region to obtain three peak areas corresponding to the β-sheet, α-helix and β-turn structures, according to Kong and Yu [16]. The percentage of each secondary structure identified in chicken breast meat for all treatments is shown in the Table 2. The percentage of α-helix structures was lower (p≤0.05) in samples from broilers scalded at T_5_ conditions, and the value was statistically similar to T_1_. Samples from T_4_ treatment exhibited the highest α-helix percentage, and intermediate values were observed in T_2_ and T_3_, demonstrating that in these experimental conditions, the scalding did not significantly affect the protein secondary structure. Hydrogen bonds between the carbonyl oxygen and amino hydrogen of the polypeptide chain are related to stabilization of the α-helix structure [25]. The α-helix conformation is considered an amphiphilic structure, constituted by hydrophobic and hydrophilic residues, while β-sheets and β-turns are considered more hydrophobic than α-helix structure; however, β-sheets and β-turns are thermally stable due the predominance of hydrophobic interactions [26]. When a protein solution of α-helix-type is heated and cooled, the α-helix is converted into a β-sheet [26,27]. This corroborates the observations from the T_5_ scalding treatment (58°C/90 s). In the case of T_1_ that also exhibited lower α-helix percentage despite the use of a lower scalding temperature (54°C), the exposure time was higher (210 s), which could have contributed to the conversion from α-helix to β-sheet. The 210 s time applied in T_1_ was necessary because at this temperature, the defeathering of the carcasses and the removal of the cuticle layer were impaired and required a longer exposure time. This result confirmed that beyond temperature, the exposure time of scalding can also affect the chicken breast meat properties.

In relation to the β-turn percentages, the values were higher in T_5_ samples, differing from the T_3_ samples that exhibited the lowest value (p≤0.05). T_1_, T_2_, and T_4_ samples were statistically similar to both of the treatments cited above (p>0.05). The β-turn formation is related to the 180° inversion of the polypeptide chain involved in β-sheet formation [26], and the increase of β-turn formation has been related to temperature increase [27]; therefore, these considerations explain the high proportion of β-turns in T_5_.

### Fatty acid profile

The fatty acid profiles of the chicken breast meat from the different scalding treatments are shown in Table 3. The oleic acid (18:1n-9*c*) percentage was lower in the samples from T_5_ than in those from T_4_ and T_3_. These results were statistically similar to T_1_ and T_2_. The same was observed for the sum of monounsaturated fatty acids (MUFA). The variation of oleic acid among the treatments could be related to lipid oxidation [28] caused by the heating of the scalding conditions. The percentages of eicosadienoic acid (20:2), arachidonic acid (20:4n-6) and docosahexaenoic acid (22:6n-3) were higher in T_5_ than in samples from other experimental conditions (p≤0.05). Changes in the fatty acid profile of chicken breast meat are related to phospholipase A_2_ (PLA_2_) activity, enzymes which catalyzes the hydrolysis of cellular membrane phospholipids [21,29], releasing unsaturated fatty acid at the sn-2 position [30]. It has also been related that PLA_2_ activity is increased by heating, where the optimal activity of PLA_2_ ranging from 40°C to 50°C was reported [31], and this enzyme has activity increased in minutes with temperature increase from 25°C to 35°C [32]. These polyunsaturated fatty acids (PUFA) are present in the phospholipid cellular membrane, in particular arachidonic acid [28]. Therefore, in the present study, the cellular membrane collapse could have been favored in the hard-scald method (T_5_, 58°C/90 s) with PUFA release. These considerations can also explain the variation observed in the n-3 sum among the treatments. However, the PUFA sum did not differ among the treatments (p>0.05). The results reported are relevant since the fatty acid profile for chicken breast meat from different scalding treatment have not yet been reported in the literature.

## CONCLUSION

The increase of temperature from 54°C to 58°C negatively affected the lightness and protein functionality of the chicken breast meat fillets 24 h *post-mortem*. Protein secondary structures showed conformational changes, mainly when a high temperature and a short time or a low temperature and a long time were combined. In the fatty acid profile, a lower MUFA sum and higher percentages of eicosadienoic, araquidonic, and docosahexaenoic acids were observed with temperature increase, suggesting oxidation and the collapse of the phospholipid cellular membrane. Intermediary combinations of time and temperature in the scalding procedure were demonstrated to avoid negative changes in the chicken meat quality parameters.

## Figures and Tables

**Table 1:** Analytical determinations of chicken breast meat and the yields of eviscerated and chilled carcass, and deboned chicken breast from broiler carcasses submitted to different scalding treatments (mean value±standard deviation, n = 270)

Parameters	Scalding treatments					p-value

	T1	T2	T3	T4	T5	

	54°C/210 s	55°C/180 s	56°C/150 s	57°C/120 s	58°C/90 s	
L*_ventral_	53.72^d^±1.55	54.83^c^±1.38	56.44^b^±1.84	57.27^a,b^±2.51	57.55^a^±1.92	<0.01*
a*_ventral_	−2.25^a,b^±0.46	−2.13^a^±0.46	−2.09^a^±0.53	−2.46^b^±0.49	−2.33^a,b^±0.58	<0.01*
b*_ventral_	10.19^b^±1.12	10.33^a,b^±0.86	10.51^a,b^±0.93	10.69^a,b^±1.10	10.80^a^±0.82	<0.01*
L*_external_	60.11^c^±1.47	61.05^b^±1.66	62.05^a^±1.95	62.68^a^±1.51	62.69^a^±1.68	<0.01*
a*_external_	−2.81^a,b^±0.50	−2.75^b^±0.52	−2.93^a,b^±0.58	−3.02^a^±0.47	−3.01^a^±0.39	0.02*
b*_external_	10.66±0.69	10.67±0.85	10.76±0.69	10.74±0.19	10.82±0.75	0.80
pH	5.89±0.10	5.84±0.08	5.86±0.11	5.87±0.12	5.86±0.11	0.10
WHC (%)	68.30±1.98	68.60±2.34	68.82±1.94	68.91±2.01	68.67±2.68	0.64
WB (N)	10.16±1.74	10.09±1.57	9.73±1.75	9.98±2.01	9.56±1.52	0.26
Moisture	74.35±0.68	74.26±0.56	74.41±0.80	74.52±0.64	74.42±0.59	0.39
Protein	23.66±0.74	23.69±0.74	23.69±0.69	23.50±0.75	23.65±0.64	0.58
Lipid	2.09±0.52	2.11±0.46	1.91±0.57	1.93±0.51	1.97±0.41	0.20
M/P ratio	3.15±0.12	3.14±0.12	3.12±0.18	3.18±0.12	3.15±0.11	0.22
Yield1) (%)	74.17±1.63	74.33±5.66	74.66±2.32	74.48±2.41	74.84±2.39	0.36
Yield2) (%)	80.63±2.57	79.85±6.72	80.70±2.38	79.62±3.09	79.14±2.84	0.40
Yield3) (%)	24.13±1.76	24.27±1.74	24.03±1.83	24.34±2.01	24.18±1.91	0.95
PD (%)	26.02^c^±4.64	25.00^c^±6.10	26.21^c^±5.77	31.80^b^±6.76	32.59^a^±7.52	<0.01*
EC	15.44^a^±1.59	15.72^a^±1.46	15.59^a^±1.57	14.42^b^±1.25	14.46^b^±1.17	<0.01*

WHC, water holding capacity; WB, Warner-Bratzler shear force; M/P ratio, moisture/protein ratio; PD, protein denaturation; EC, emulsification capacity (expressed in x mL of oil for 1 g of sample).

1) Eviscerated carcass yield.

2) Chilled carcass yield.

3) Deboned chicken breast yield.

* Different letters in the same row indicate significant difference by the Tukey test (p≤0.05).

**Table 2:** Comparison of protein secondary structures (%) in chicken breast meat submitted to different scalding conditions, determined by Fourier-transform infrared attenuated reflectance mode (FTIR-ATR) spectroscopy with self-deconvolution (mean value±standard deviation, n = 15)

Secondary structures (wave number cm^−1^)	Scalding treatments					p-value

	T1	T2	T3	T4	T5	

	54°C/210 s	55°C/180 s	56°C/150 s	57°C/120 s	58°C/90 s	
β-sheet (1624)	65^a^±2	49^a,b^±5	49^a,b^±6	37^b^±4	61^a^±4	0.01*
α-helix (1643)	26^b^±3	43^a,b^±7	46^a,b^±5	57^a^±5	22^b^±5	0.02*
β-turn (1689)	9^a,b^±5	8^a,b^±2	5^b^±1	6^a,b^±1	17^a^±1	0.05*

* Different letters in the same row indicate significant difference by the Tukey test (p≤0.05).

**Table 3:** Total fatty acid composition (the main fatty acids are expressed as a % of the total fatty acids) in chicken breast meat from broilers submitted to different scalding treatments (mean value±standard deviation, n = 45)

Fatty acid	Scalding treatments					p-value

	T1	T2	T3	T4	T5	

	54°C/210 s	55°C/180 s	56°C/150 s	57°C/120 s	58°C/90 s	
14:0 (myristic)	0.47±0.04	0.44±0.03	0.46±0.03	0.47±0.02	0.44±0.05	0.10
14:1n-5 (myristoleic)	0.08±0.02	0.08±0.02	0.09±0.01	0.09±0.02	0.08±0.02	0.54
16:0 (palmitic)	21.91±1.2	21.27±0.98	21.90±1.32	22.07±1.40	21.68±1.22	0.48
16:1n-7 (palmitoleic)	3.18±0.60	3.32±0.65	3.42±0.35	3.41±0.61	3.0±0.66	0.35
18:0 (stearic)	7.27±0.49	7.16±0.48	7.02±0.54	7.18±0.33	7.51±0.63	0.31
18:1n-9*c* (oleic)	31.60^a,b^±0.99	31.33^a,b^±0.84	31.82^a^±0.91	31.93^a^±1.10	30.54^b^±1.29	0.02*
18:2n-6*c* (linoleic)	28.96±2.37	29.66±1.99	28.90±1.55	28.34±2.96	28.76±2.37	0.54
18:3n-6 (γ-linolenic)	0.20±0.04	0.20±0.03	0.20±0.04	0.17±0.03	0.20±0.02	0.27
20:0 (arachidic)	0.09±0.01	0.09±0.01	0.10±0.01	0.09±0.01	0.09±0.01	0.11
18:3n-3 (α-linolenic)	1.83±0.23	1.89±0.29	1.81±0.23	1.81±0.35	1.73±0.25	0.17
20:1n-9 (gadoleic)	0.26±0.01	0.26±0.02	0.26±0.02	0.27±0.01	0.26±0.01	0.53
20:2 (eicosadienoic)	0.40^b^±0.04	0.42^b^±0.03	0.40^b^±0.09	0.41^b^±0.05	0.49^a^±0.03	<0.01*
20:3n-6 (DHGL)	0.53±0.09	0.52±0.07	0.48±0.10	0.54±0.14	0.57±0.10	0.13
22:0 (docosanoic)	0.02±0.01	0.02±0.01	0.02±0.01	0.02±0.01	0.02±0.01	0.21
20:4n-6 (AA)	2.82^b^±0.37	2.89^b^±0.39	2.70^b^±0.53	2.81^b^±0.61	3.97^a^±0.52	<0.01*
20:5n-3 (EPA)	0.06±0.02	0.07±0.04	0.06±0.02	0.06±0.03	0.08±0.02	0.42
22:6n-3 (DHA)	0.32^b^±0.08	0.39^b^±0.05	0.34^b^±0.08	0.33^b^±0.06	0.53^a^±0.15	<0.01*
∑SFA	29.75±1.48	28.98±1.19	29.50±1.87	29.83±1.59	29.74±1.59	0.67
∑MUFA	35.12^a,b^±1.56	34.98^a,b^±1.44	35.60^a^±1.09	35.69^a^±1.57	33.92^b^±1.89	0.04*
∑PUFA	35.13±2.33	36.03±2.29	34.90^a^±2.08	34.48^a^±2.64	36.33^a^±2.83	0.10
∑n-6	32.51^a^±2.16	33.26^a^±2.03	32.29±1.78	31.87±2.41	33.50±2.49	0.14
∑n-3	2.22^b,c^±0.21	2.36^a^±0.26	2.21^c^±0.23	2.20^c^±0.27	2.34^a,b^±0.34	0.03*
n-6/n-3	14.70±0.78	14.19±0.85	14.69±0.82	14.57±1.06	14.48±1.14	0.23

DHGL, dihomo-γ-linolenic acid; AA, arachidonic acid; EPA, eicosapentaenoic acid; DHA, docosahexaenoic acid; SFA, saturated fatty acids; MUFA, monounsaturated fatty acids; PUFA, polyunsaturated fatty acids.

* Different letters in the same row indicate significant difference by Tukey test (p≤0.05).

## References

[1] Cason JA, Buhr RJ, Hinton A (2001). Unheated water in the first tank of a three-tank broiler scalder. Poult Sci.

[2] Sams AR, McKee SR, Owens CM, Alvarado CZ, Sams AR (2010). First processing: slaughter through chilling. Poult meat process.

[3] Buhr RJ, Walker JM, Bourassa DV, Caudill AB, Kiepper BH, Zhuang H (2014). Impact of broiler processing scalding and chilling profiles on carcass and breast meat yield. Poult Sci.

[4] McKee SR, Townsend JC, Bilgili SF (2008). Use of a scald additive to reduce levels of *Salmonella* Typhimurium during poultry processing. Poult Sci.

[5] Jeong JY, Janardhanan KK, Booren AM, Karcher DM, Kang I (2011). Moisture content, processing yield, and surface color of broiler carcasses chilled by water, air, or evaporative air. Poult Sci.

[6] Bowker BC, Zhuang H, Buhr RJ (2014). Impact of carcass scalding and chilling on muscle proteins and meat quality of broiler breast fillets. LWT - Food Sci Technol.

[7] Zhuang H, Bowker BC, Jeff Buhr R, Bourassa DV, Kiepper BH (2013). Effects of broiler carcass scalding and chilling methods on quality of early-deboned breast fillets. Poult Sci.

[8] Tornberg E (2005). Effects of heat on meat proteins - Implications on structure and quality of meat products. Meat Sci.

[9] Olivo R, Soares AL, Ida EI (2001). Dietary vitamin E inhibits poultry PSE and improves meat function properties. J Food Biochem.

[10] Hamm R (1960). Biochemistry of meat hydration. Adv Food Res.

[11] Olivo R, Shimokomaki M (2002). Carnes: no caminho da pesquisa.

[12] Qiao M, Fletcher DL, Smith DP, Northcutt JK (2001). The effect of broiler breast meat color on pH, moisture, water-holding capacity, and emulsification capacity. Poult Sci.

[13] Swatland HJ (1995). Optical properties of meat. On-line evaluation meat.

[14] Bligh EG, Dyer WJ (1959). A rapid method of total lipid extraction and purification. Can J Biochem Physiol.

[15] International Organization for Standardization (1978). ISO 5509:animal and vegetable fats and oils: preparation of methyl esters of fatty acids.

[16] Kong J, Yu S (2007). Fourier transform infrared spectroscopic analysis of protein secondary structures. Acta Biochim Biophys Sin (Shanghai).

[17] AOAC (2007). Fat, moisture and protein in meat and meat products. FOSS foodscan near-infrared (NIR) spectrophotometer with FOSS artificial neural network (ANN) calibration model and associated. J AOAC Int.

[18] AOAC (2005). Official methods of analysis of the Association of Official Analytical Chemists.

[19] Jiang H, Yoon SC, Zhuang H, Wang W (2017). Predicting color traits of intact broiler breast fillets using visible and near-infrared spectroscopy. Food Anal Methods.

[20] Kato T, Barbosa CF, Ida EI, Soares AL, Shimokomaki M, Pedrao MR (2013). Broiler chicken PSE (pale, soft, exudative) meat and water release during chicken carcass thawing and Brazilian legislation. Braz Arch Biol Technol.

[21] da Silva-Buzanello RA, Schuch AF, Nunes Nogues DR (2018). Physicochemical and biochemical parameters of chicken breast meat influenced by stunning methods. Poult Sci.

[22] Wilhelm AE, Maganhini MB, Hernández-Blazquez FJ, Ida EI, Shimokomaki M (2010). Protease activity and the ultrastructure of broiler chicken PSE (pale, soft, exudative) meat. Food Chem.

[23] Strasburg G, Xiong YL, Chiang W (2010). Physiology and chemistry of edible muscle tissues. Química Aliment Fennema.

[24] Brazil. Instrução Normativa no 32 de 03 de dezembro de 2010 (2010). Parameters for evaluation of the total water content in cold and frozen chicken cuts.

[25] Liu R, Zhao SM, Xiong SB, Xie BJ, Qin LH (2008). Role of secondary structures in the gelation of porcine myosin at different pH values. Meat Sci.

[26] Damodaran S, Damodaran S, Parkin KL, Fennema OR (2010). Amino acids, peptides and proteins. Química aliment fennema.

[27] Xu XL, Han MY, Fei Y, Zhou GH (2011). Raman spectroscopic study of heat-induced gelation of pork myofibrillar proteins and its relationship with textural characteristic. Meat Sci.

[28] Murakami M, Kudo I (2002). Phospholipase A2. J Biochem.

[29] Soares AL, Ida EI, Miyamoto S, Hernandez-Blazque FJ, Olivo R, Pinheiro JW (2003). Phospholipase A2 activity in poultry PSE, pale, soft, exudative, meat. J Food Biochem.

[30] Lambert IH, Nielsen JH, Andersen HJ, ørtenblad N (2001). Cellular model for induction of drip loss in meat. J Agric Food Chem.

[31] Morgado MAP, Cabral JMS, Prazeres DMF (1995). Hydrolysis of lecithin by phospholipase A2 in mixed reversed micelles of lecithin and sodium dioctyl sulphosuccinate. J Chem Technol Biotechnol.

[32] Belhadj Slimen I, Najar T, Ghram A, Abdrrabba M (2016). Heat stress effects on livestock: molecular, cellular and metabolic aspects, a review. J Anim Physiol Anim Nutr (Berl).

